# Differential diagnosis of benign and lung adenocarcinoma presenting as larger solid nodules and masses based on multiscale CT radiomics

**DOI:** 10.1371/journal.pone.0309033

**Published:** 2024-10-04

**Authors:** Jiayue Xie, Yifan He, Siyu Che, Wenjing Zhao, Yuxin Niu, Dongxue Qin, Zhiyong Li

**Affiliations:** 1 Department of Radiology, The First Affiliated Hospital of Dalian Medical University, Dalian, Liaoning Province, China; 2 Department of Radiology, The Second Hospital of Dalian Medical University, Dalian, Liaoning Province, China; University of Pisa, ITALY

## Abstract

**Purpose:**

To develop a better radiomic model for the differential diagnosis of benign and lung adenocarcinoma lesions presenting as larger solid nodules and masses based on multiscale computed tomography (CT) radiomics.

**Materials and methods:**

This retrospective study enrolled 205 patients with solid nodules and masses from Center 1 between January 2010 and February 2022 and Center 2 between January 2019 and February 2022. After applying the inclusion and exclusion criteria, we retrospectively enrolled 165 patients from two centers and assigned them to the training dataset (n = 115) or the test dataset (n = 50). Radiomics features were extracted from volumes of interest on CT images. A gradient boosting decision tree (GBDT) was used for data dimensionality reduction to perform the final feature selection. Four models were developed using clinical data, conventional imaging features and radiomics features, namely, the clinical and image model (CIM), the plain CT radiomics model (PRM), the enhanced CT radiomics model (ERM) and the combined model (CM). Model performance was evaluated to determine the best model for identifying benign and lung adenocarcinoma presenting as larger solid nodules and masses.

**Results:**

In the training dataset, the areas under the curve (AUCs) for the CIM, PRM, ERM, and CM were 0.718, 0.806, 0.819, and 0.917, respectively. The differential diagnostic capability of the ERM was better than that of the PRM and the CIM. The CM was optimal. Intermediate and junior radiologists and respiratory physicians achieved improved obviously diagnostic results with the radiomics model. The senior radiologists showed slight improved diagnostic results after using the radiomics model.

**Conclusion:**

Radiomics may have the potential to be used as a noninvasive tool for the differential diagnosis of benign and lung adenocarcinoma lesions presenting as larger solid nodules and masses.

## Introduction

Larger solid nodules and masses in the lungs are common and fundamental imaging manifestations of many diseases of the lung. Malignant larger solid nodules and masses are more malignant, metastasize earlier, and have a worse prognosis [1–4]. In addition, many guidelines have been developed to support management decisions, which include the lesion size as a key risk factor [5]. The treatment of an individual with a solid pulmonary nodule 8 mm or larger is based on the estimated probability of malignancy [6]. Therefore, the differential diagnosis of benign and lung adenocarcinoma lesions presenting as larger solid nodules and masses is vital to increase the correct diagnosis rate and improve patient prognosis.

Many researchers have recognized that morphological features on CT images do not provide a complete picture for diagnosis and that a large amount of unseen, high-dimensional, valuable data are hidden in the images [7–11]. Radiomics is a promising tool for the identification of malignant pulmonary nodules or masses [12–21].

Most studies are based on noncontrast-enhanced CT images for benign and malignant differential diagnosis of lung nodules or masses [22, 23]. Contrast-enhanced CT images usually reflect the blood supply to the lesion, and enhanced attenuation helps to differentiate between benign and malignant lesions. Some researchers have begun to explore the value of contrast-enhanced CT images [24–27]. Qin Liu et al. [26] reported that the radiomics nomogram of enhanced CT could be used to predict lung adenocarcinoma. In addition, contrast-enhanced CT is more useful in differentiating pulmonary tuberculosis from pulmonary adenocarcinoma via radiomics [27].

In this study, we used plain and enhanced chest CT imaging radiomics combined with clinical data and conventional CT features to construct a predictive model for the differential diagnosis of benign and lung adenocarcinoma lesions presenting as larger solid nodules and masses to guide clinical decision-making.

## Materials and methods

This retrospective study was approved by the ethics committees of the two research centers, and the requirement for informed consent was waived. The ethical approval number is PJ-KS-KY-2021-291. Clinical, imaging and radiomics data are provided in **S1–S3 Tables**.

### Patients

Data were accessed for research purposes beginning on January 1^st^, 2023. The authors had no access to information that could identify individual participants during or after data collection.

**Fig 1:**
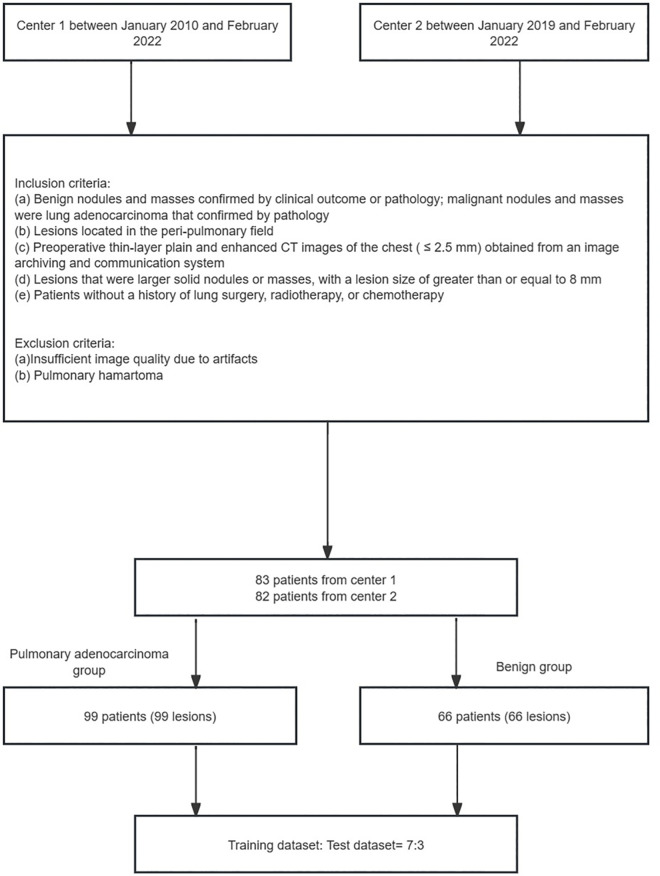
Flow chart of the lesion selection and grouping.

This retrospective study enrolled 205 patients with solid nodules and masses from Center 1 between January 1^st^, 2010, and February 28^th^, 2022, and Center 2 between January 1^st^, 2019, and February 28^th^, 2022. All patients underwent chest CT examination before surgery (**Fig 1**).

We searched patients with the following inclusion criteria: (a) benign nodules and masses confirmed by clinical outcome or pathology; malignant nodules and masses confirmed by pathology; (b) lesions located in the peripulmonary field; (c) preoperative thin-layer plain and enhanced CT images of the chest (≤ 2.5 mm) obtained from an image archiving and communication system; (d) lesions that were larger solid nodules and masses, with a lesion size greater than or equal to 8 mm; and (e) patients without a history of lung surgery, radiotherapy, or chemotherapy. The exclusion criteria for this study included (a) insufficient image quality due to artifacts; and (b) pulmonary hamartoma (pulmonary hamartoma was excluded due to the presence of fat and low enhancement within it).

After applying the inclusion and exclusion criteria, we recruited 83 patients at Study Center 1 and 82 patients at Study Center 2. Finally, 165 patients (99 patients in the pulmonary adenocarcinoma group and 66 patients in the benign group) were included in this study. We included 115 patients in the training dataset at a 7:3 ratio, and we included 50 patients in the test dataset (**Fig 1**). Each patient corresponded to one lesion. Benign larger nodules and masses consisted of 3 lesions with effective clinical treatment and 63 pathologically confirmed lesions. The three benign lesions were significantly reduced or completely absorbed after anti-inflammatory therapy at the second examination. After 76 days and 4 years of follow-up, the lesions of 2 benign lesions were completely absorbed. After 7 days of follow-up, 80% of the third benign lesion was absorbed. Malignant larger nodules and masses were pathologically confirmed to be lung adenocarcinoma.

### CT image acquisition

The chest CT images of Center 1 were acquired using an Optima CT660/Discovery CT750 HD/LightSpeed Plus/LightSpeed16/Revolution CT (General Electric), uCT 760 (United Imaging Healthcare Technology), and Brilliance 16P (Philips). The chest CT images of Center 2 were acquired using an SOMATOM Drive/SOMATOM Definition (Siemens) and iCT 256 (Philips). The patient was placed in the supine position, scanned from the lung apex to the lower border of the costal diaphragmatic angle, and scanned with deep inspiratory breath-hold imaging. The tube voltage ranged from 120–140 kV, and the tube current ranged from 140–630 mAs or was automatically adjusted according to the patient’s weight. The reconstruction thickness was 1.0–2.5 mm, and the reconstruction interval was 1.0–2.5 mm. The contrast agent was injected at a rate of 2.5–3.0 mL/s. Enhanced CT scans were performed 55–60 s after contrast agent injection.

### Clinical information and evaluation of CT images

The CT images were analyzed independently by two chest radiologists (with 5 years of chest imaging experience, Jiayue Xie; and 2 years of chest imaging experience, Yuxin Niu) experienced in examining chest images; these radiologists had no knowledge of the final pathologic diagnosis or medical history. The radiologists reviewed each patient’s chest CT images via RadiAnt DICOM Viewer 2021.2 (64-bit) software (Poland). When there was disagreement, the opinion of the highest-ranking radiologist was considered (29 years of imaging experience, Zhiyong Li).

CT images of the lung (1500 HU window width; -600 HU window level) and mediastinum (400 HU window width; 40 HU window level) windows were analyzed. The parameters examined in the CT images were as follows: (1) max diameter (the longest diameter at the maximum level of the axial lesion); (2) mean diameter (long and short diameters in the largest cross-section, to compute the mean); (3) location (lower lobe; upper and middle lobes); (4) spiculation (absent, present); (5) lobulation (absent, present); (6) cavity (absent, present); (7) pleural traction (absent, present); (8) air bronchogram (absent, present); and (9) calcification (absent, present). The sex, age and smoking status of each patient were recorded.

### Lesion segmentation and radiomics feature extraction

The CT images of each patient included the lung window and mediastinum window. The mediastinum window was used to extract the radiomics features. The region of interest (ROI) of each CT image was segmented by one doctor (5 years of diagnostic chest imaging experience, Jiayue Xie) via the published 3D Slicer software version 5.0.3 (http://slicer.org/). The CT images of each patient were manually segmented in the visible area of the lesion. The resampling process was performed prior to feature extraction, and the voxel size was adjusted to 1 mm × 1 mm × 1 mm. The 3D slicing software automatically extracts 107 radiomics features from the volumes of interest, and the radiomics features are subdivided into the following categories: first-order statistical features, shape-based features, and statistically-based texture features.

Thirty lesions were randomly selected from 165 lesions for intra- and intergroup consistency testing one month after the initial segmentation of the ROIs. Chest radiologist A (5 years of chest imaging experience, Jiayue Xie) and chest radiologist B (2 years of chest imaging experience, Yuxin Niu) evaluated the intra- and intergroup consistency for 30 lesions. The final pathological results of the 30 lesions were unknown to the radiologists at the time of assessment. Inter- and intraclass correlation coefficients (ICCs) were used to assess the inter- and intraobserver agreement of feature extraction. After the ICCs of each feature were calculated, an ICC>0.75 indicated that the feature had good or excellent consistency.

### Feature selection and model construction

First, the radiomics feature data were processed by normalized ICCs (values < 0.5 indicated poor reliability, values between 0.5 and 0.75 indicated moderate reliability, values between 0.75 and 0.9 indicated good reliability, and values > 0.90 indicated excellent reliability) [28]. Second, the radiomics features in the training dataset were screened via correlation analysis (if the average correlation coefficient of a feature with other features exceeded 0.8, the feature was rejected). Afterward, the screening of features was continued via the gradient boosting decision tree (GBDT). Differences in clinical and conventional imaging features between the benign and malignant groups were compared via univariate and multivariate analyses. Using the features selected above, we used logistic regression (LR) to build four models. We developed and compared four models, including the clinical and image model (CIM), the plain CT radiomics model (PRM), the enhanced CT radiomics model (ERM) and the combined model (CM). The CM was formed by a combination of the CIM, the PRM, and the ERM. After identifying the best LR model, we used its modeling features to build a support vector machine (SVM) model, a random forest (RF) model, a decision tree (DT) model, and a k-nearest neighbor (KNN) model.

### Evaluation of model performance

**Fig 2:**
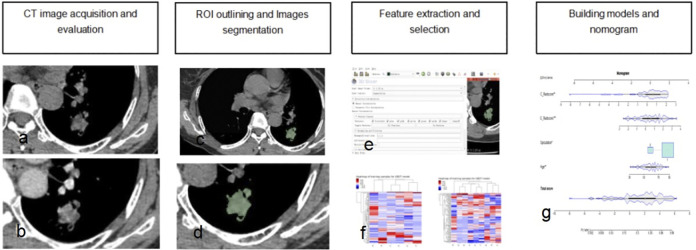
Flow chart displaying the process of building the radiomics-based model. a: Plain period of the lesion. b: Venous phase of the lesion. c-d: ROI outlining and image segmentation. e-f: Feature extraction and selection. g: Developed combined model nomogram. ROI, region of interest.

Receiver operating characteristic (ROC) curve analysis was used to individually evaluate the value of the four models in the training and test datasets. We individually calculated the area under the curve (AUC), sensitivity, specificity and accuracy. The following calculation formulas for the corresponding indexes were use: *Sensitivity* = *TP*/(*TP*+*FN*); *Sensificity* = *TN*/(*TN*+*FP*); and *Accuracy* = (*TP*+*TN*)/(*TP*+*TN*+*FP*+*FN*). The abbreviations for true positive, false positive, true negative and false negative in the formulas are TP, FP, TN, and FN, respectively. The calibration curve and the Hosmer–Lemeshow test were used to judge how well each model fit the ideal model. Decision curve analysis (DCA) was performed in accordance with the net benefit and the corresponding threshold probability to assess the clinical practicality of the models. A flow chart displaying the process of building the radiomics-based model in this study is shown in **Fig 2.**

We invited a senior radiologist (senior radiologist A, Zhiyong Li, with 29 years of chest imaging experience), an intermediate radiologist (intermediate radiologist B, Ruyi Bao, with 15 years of chest imaging experience), a junior radiologist (junior radiologist C, Siyu Che, with 5 years of chest imaging experience), an intermediate respiratory physician (intermediate respiratory physician D, Yadong Zhao, with 16 years of chest experience) and a junior respiratory physician (junior respiratory physician E, Meihong Liu, with 4 years of chest experience) to independently diagnose all lesions. Then, using the radiomics model, all five doctors were asked to diagnose the lesions again. The diagnostic efficiency of the radiomic model was evaluated by 5 doctors.

### Statistical analysis

All the statistical analyses were performed using R 4.1.1 (https://cran.r-project.org/). The goodness of fit was calculated using the Hosmer–Lemeshow test. ROC analyses were performed via "pROC". DCA was performed using "dca.r".

Categorical variables (e.g., sex) were compared via the Pearson chi-square test and Fisher’s exact test, whereas continuous variables (e.g., age) that conformed to a normal distribution were compared using the t test. Continuous variables that did not fit a normal distribution were compared using the Mann‒Whitney U test. All the statistical analyses were two-tailed. A p value less than 0.05 was considered statistically significant.

## Results

### Analysis of clinical data and conventional imaging features

The mean diameter of larger solid nodules and masses ranged from 1.1 cm to 4.6 cm. The 66 benign lesions included tuberculous nodules, inflammatory nodules, inflammatory granulomas, pulmonary cryptococcosis, pulmonary aspergillosis, pulmonary *Candida albicans*, pulmonary abscesses, and sclerosing pneumocytoma.

**Fig 3:**
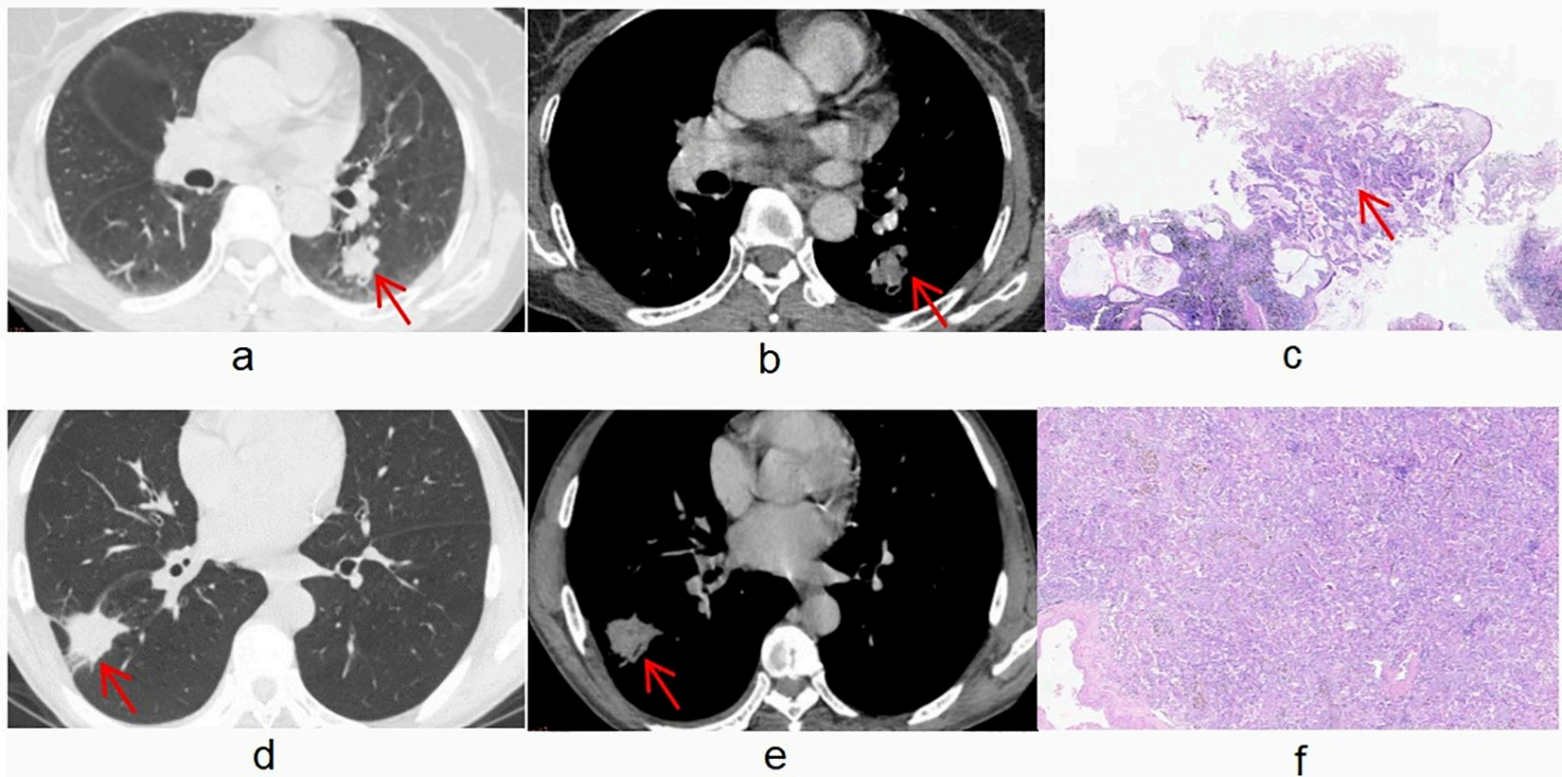
Chest CT and pathological diagnosis of the pulmonary larger solid nodules and masses (a, b, c, d, e, and f). Lesions are shown by arrows. Chest CT images (**a and b**) of a 61-year-old female patient revealed a solid nodule in the lower lobe of the left lung, and this nodule was pathologically (**c**) diagnosed as pulmonary adenocarcinoma. Chest CT images (**d and e**) of a 60-year-old male patient revealed a solid mass in the lower lobe of the right lung, and this mass was pathologically (**f**) diagnosed as organizing pneumonia.

The training dataset (115 patients) included 46 patients with benign lesions and 69 patients with malignant lesions. The test dataset included 20 benign patients and 30 malignant patients. No significant differences were found between the training dataset and the test dataset in terms of clinical or imaging features (**Table 1**). Using univariate and multivariate logistic regressions in the training dataset, age (odds ratio (OR) 1.069; 95% confidence interval (CI) 1.023–1.116; p = 0.003) and spiculation (OR 5.515; 95% CI 1.544–19.702; p = 0.009) were identified as independent predictors (**S4 Table**). Examples of organizing pneumonia and pulmonary adenocarcinoma lesions are shown in **Fig 3**.

**Table 1:** Basic information about the patients in the training and test datasets.

	Training dataset			Test dataset			P
	benign	malignant	p	benign	malignant	p	
	N = 46	N = 69		N = 20	N = 30		
Age	54.6±10.8	61.5±10.2	0.001*	57.0 [49.8;62.2]	62.0 [59.0;67.8]	0.006*	0.671
Sex			0.016*			0.225	0.873
Famale	14 (30.4%)	38 (55.1%)		7 (35.0%)	17 (56.7%)		
Male	32 (69.6%)	31 (44.9%)		13 (65.0%)	13 (43.3%)		
Smoking status			0.032*			0.520	0.354
Never smoked	25 (54.3%)	52 (75.4%)		10 (50.0%)	19 (63.3%)		
Ex-or current smoker	21 (45.7%)	17 (24.6%)		10 (50.0%)	11 (36.7%)		
Max diameter	2.70 [1.90;3.38]	2.40 [1.80;3.00]	0.178	2.50 [2.30;3.32]	2.45 [1.90;3.08]	0.289	0.918
Mean diameter	2.30 [1.70;2.98]	2.00 [1.50;2.80]	0.178	2.4±0.7	2.2±0.7	0.456	0.790
Location			1.000			0.951	0.190
Lower	20 (43.5%)	31 (44.9%)		7 (35.0%)	9 (30.0%)		
Upper and middle	26 (56.5%)	38 (55.1%)		13 (65.0%)	21 (70.0%)		
Spiculation			0.024*			0.047*	1.000
Absent	12 (26.1%)	6 (8.70%)		6 (30.0%)	2 (6.67%)		
Present	34 (73.9%)	63 (91.3%)		14 (70.0%)	28 (93.3%)		
Lobulation			0.167			0.289	0.717
Absent	8 (17.4%)	5 (7.25%)		3 (15.0%)	1 (3.33%)		
Present	38 (82.6%)	64 (92.8%)		17 (85.0%)	29 (96.7%)		
Cavity			0.397			0.416	1.000
Absent	38 (82.6%)	62 (89.9%)		16 (80.0%)	27 (90.0%)		
Present	8 (17.4%)	7 (10.1%)		4 (20.0%)	3 (10.0%)		
Pleural traction			0.539			0.641	0.778
Absent	6 (13.0%)	6 (8.70%)		1 (5.00%)	3 (10.0%)		
Present	40 (87.0%)	63 (91.3%)		19 (95.0%)	27 (90.0%)		
Air bronchogram			1.000			1.000	0.400
Absent	28 (60.9%)	43 (62.3%)		14 (70.0%)	21 (70.0%)		
Present	18 (39.1%)	26 (37.7%)		6 (30.0%)	9 (30.0%)		
Calcification			0.263			1.000	0.279
Absent	41 (89.1%)	66 (95.7%)		20 (100%)	29 (96.7%)		
Present	5 (10.9%)	3 (4.35%)		0 (0.00%)	1 (3.33%)		

Categorical variables are expressed as numbers (%); normally distributed continuous variables are expressed as the means ± standard deviations; abnormally distributed continuous variables are expressed as medians (first quartile, third quartile). *P value < 0.05.

### Radiomics feature screening

A total of 107 radiomic features were extracted. First, 18 features with an ICC < 0.75 were excluded. The consistency assessment revealed that the mean inter- and intragroup correlation coefficient values for the remaining 89 radiomics features ranged from 0.753–1.000 and 0.776–1.000, respectively. This indicated inter- and intraobserver consistency for the 89 radiomics features. After filtering by correlation analysis, 22 plain CT radiomics features and 22 enhanced CT radiomics features were retained. Finally, the GBDT algorithm selected 6 plain CT radiomics features and 9 enhanced CT radiomics features as the best features.

### Model development and comparison

**Fig 4:**
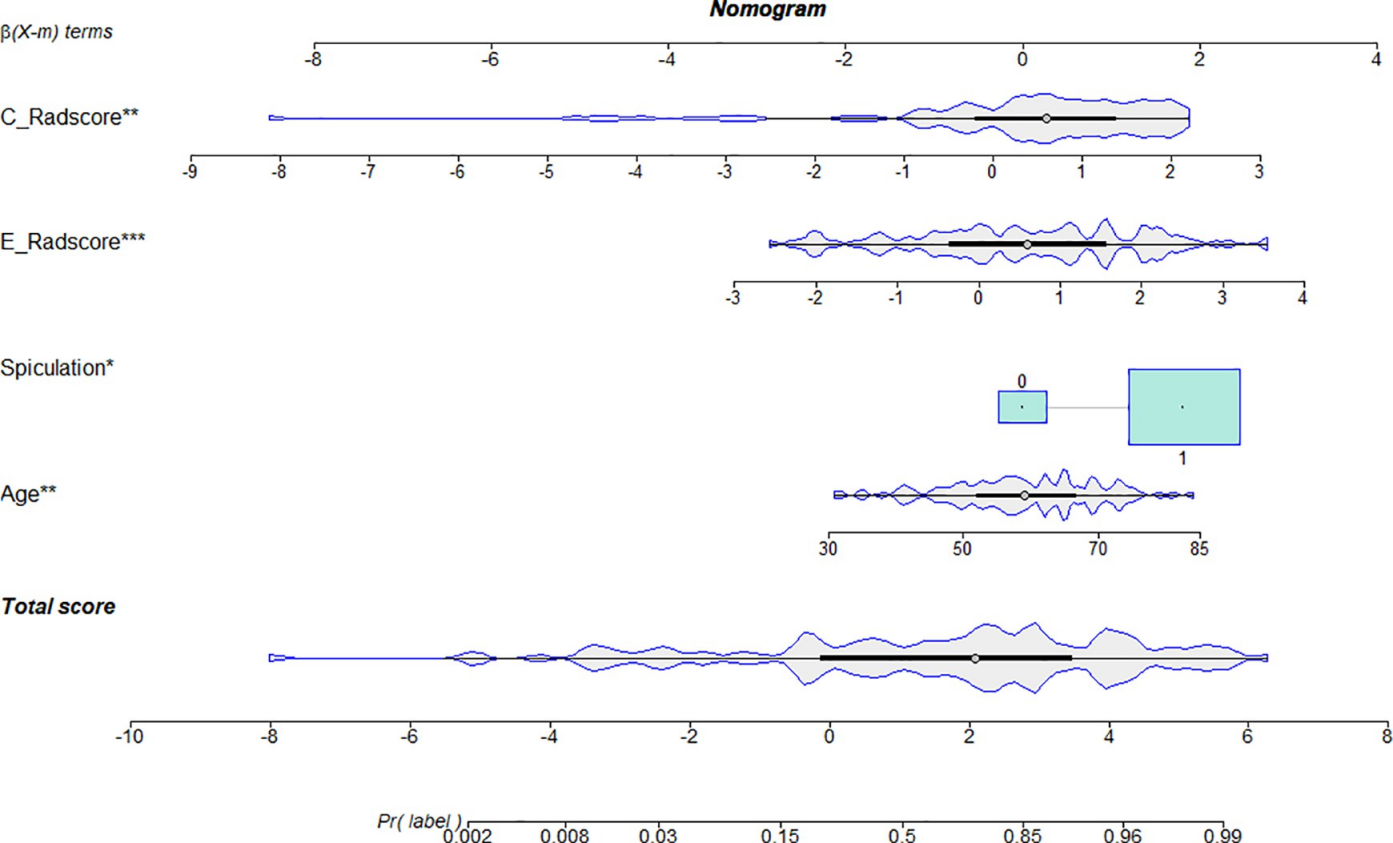
Developed CM model nomogram. *CM*, combined model.

**Fig 5:**
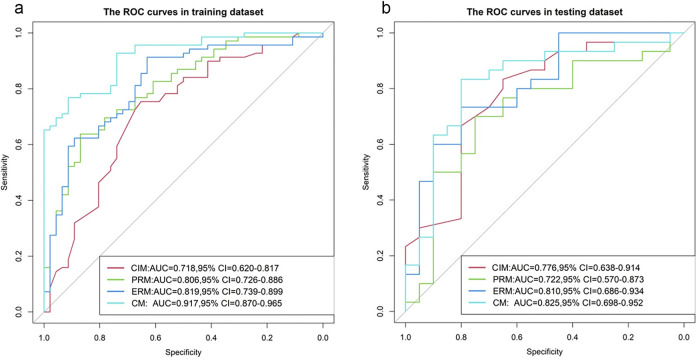
Receiver operating characteristic (ROC) curves for the four models in the training (a) and test (b) datasets. *CIM*, clinical and image model; *PRM*, plain CT radiomics model; *ERM*, enhanced CT radiomics model; *CM*, combined model; *AUC*, area under the ROC curve; *CI*, confidence interval.

Four LR models were constructed in the training set. The four models are the CIM, PRM, ERM and CM. Age and spiculation were applied to create the CIM. The model equations of the PRM and ERM are shown in **S1 Fig**. The CM was constructed by combining the CIM, the PRM and the ERM. The CM was constructed and presented as a radiomics nomogram (**Fig 4**). The ROC curves of the four models are shown in **Fig 5**. **Table 2** lists the AUC, sensitivity, specificity and accuracy of the four models. For the training and test datasets, the ERM (training dataset: AUC = 0.819; test dataset: AUC = 0.810) outperformed the CIM (training dataset: AUC = 0.718; test dataset: AUC = 0.776).

**Table 2:** Diagnostic efficacy of the four models in the training and test datasets.

Model	Training dataset				Test dataset			
	AUC (95% CI)	SEN	SPE	ACC	AUC (95% CI)	SEN	SPE	ACC
CIM	0.718(0.620, 0.817)	0.841	0.435	0.678	0.776 (0.638, 0.914)	0.900	0.500	0.740
PRM	0.806(0.726, 0.886)	0.870	0.522	0.730	0.722 (0.570, 0.873)	0.767	0.600	0.700
ERM	0.819 (0.739, 0.899)	0.855	0.652	0.774	0.810 (0.686, 0.934)	0.800	0.600	0.720
CM	0.917 (0.870, 0.965)	0.870	0.739	0.817	0.825 (0.698, 0.952)	0.833	0.800	0.820

*CIM*, clinical and image model; *PRM*, plain CT radiomics model; *ERM*, enhanced CT radiomics model; *CM*, combined model; *AUC*, area under the receiver operating characteristic curve; *CI*, confidence interval;*SEN*,sensitivity; *SPE*,specificity; *ACC*,accuracy.

For the training and test datasets, the AUC and accuracy of the ERM were greater than those of the PRM. Among all the models, the CM (training dataset: AUC = 0.917; test dataset: AUC = 0.825) was the best model.

**Fig 6:**
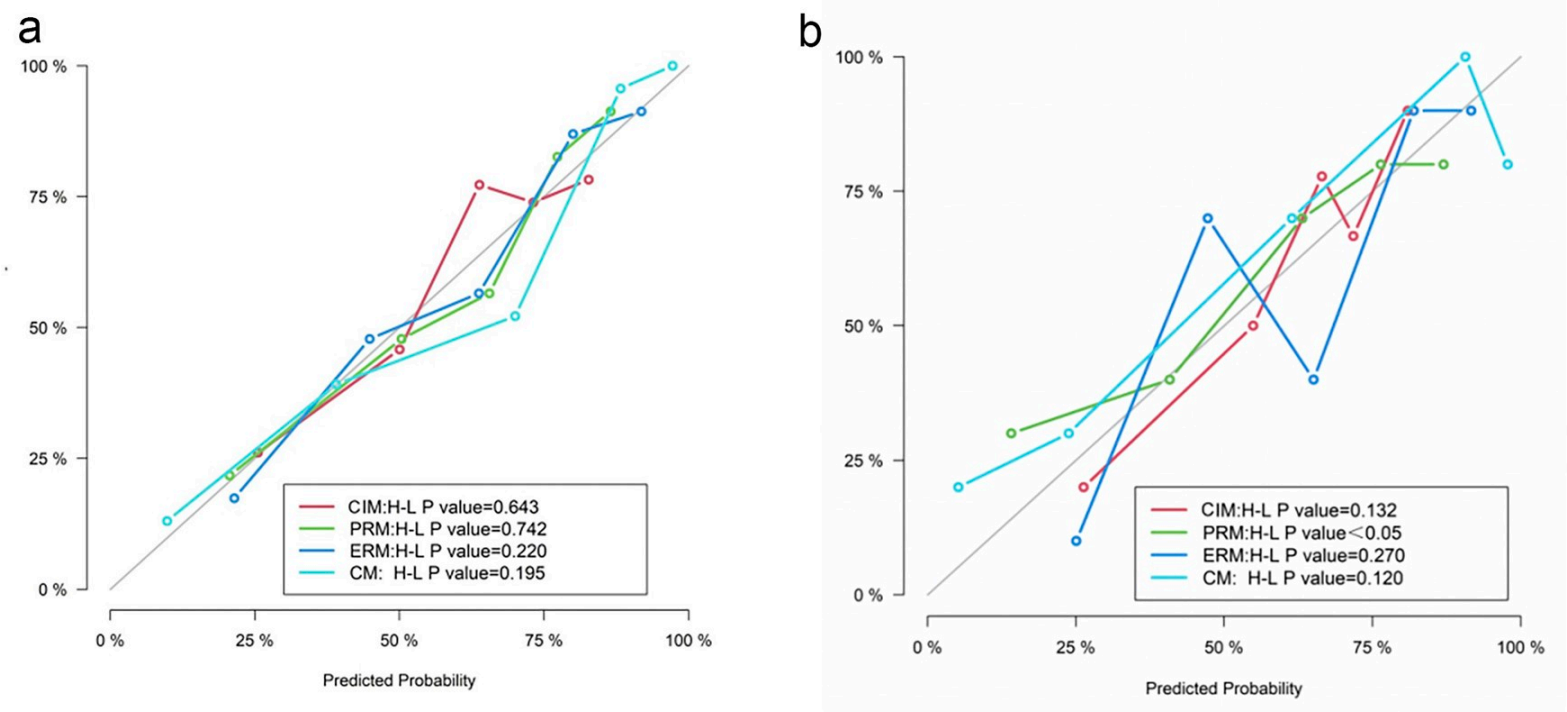
Calibration curves of the four models in the training (a) and test (b) datasets. A *P* value > 0.05 indicates that the goodness of fit is good between the model and the ideal model. *CIM*, clinical and image model; *PRM*, plain CT radiomics model; *ERM*, enhanced CT radiomics model; *CM*, combined model; *H–L*, Hosmer–Lemeshow.

**Fig 7:**
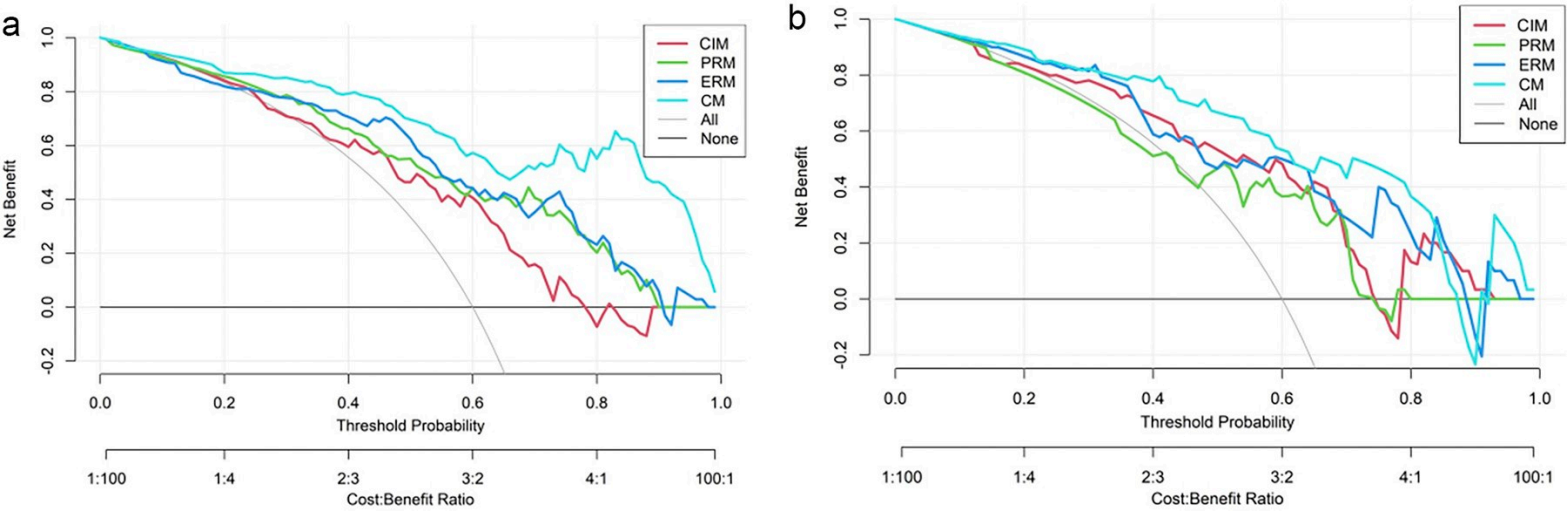
Decision curve analysis for the four models in the training (a) and test (b) datasets. “All” indicates that all lesions were diagnosed as lung adenocarcinoma. “None” indicated that all lesions were diagnosed as benign nodules and masses, and the net benefit was zero. *CIM*, clinical and image model; *PRM*, plain CT radiomics model; *ERM*, enhanced CT radiomics model; *CM*, combined model.

The calibration curves of the four models are shown in **Fig 6**. The P values of the Hosmer–Lemeshow test for the ERM and the CM were greater than 0.05 in the training and test datasets, which indicates better calibration capability. The DCA results of the four models in the training dataset and test dataset are shown in **Fig 7**. The overall net benefit of the ERM was greater than that of the PRM in most probability threshold ranges, and the overall net benefit of the CM was the greatest. The CM had the highest clinical value in differentiating between benign and lung adenocarcinoma presenting as larger solid nodules and masses.

In the test dataset, the CM (test dataset: AUC = 0.825) outperformed the intermediate and junior radiologists and intermediate and junior respiratory physicians (test dataset: AUC = 0.767, AUC = 0.733, AUC = 0.700, AUC = 0.633). The CM (test dataset: AUC = 0.825) was equivalent to that of the senior radiologist (test dataset: AUC = 0.867).

After referring to the radiomics model, the AUCs of three radiologists and two respiratory physicians improved. The AUCs for the senior, intermediate, and junior radiologists increased by 0.016, 0.050 and 0.017, respectively. The AUCs for the intermediate and junior respiratory physicians increased by 0.075 and 0.142, respectively. The diagnostic performance of the respiratory physicians improved more than that of the radiologists did (**Table 3**).

**Table 3:** Diagnostic efficacy of the five doctors independently and with the help of the radiomics model.

Doctors	AUC (95% CI)	SEN	SPE	ACC
senior radiologist A	0.867(0.757,0.976)	0.833	0.900	0.860
senior radiologist A+radiomics	0.883(0.779,0.988)	0.867	0.900	0.880
intermediate radiologist B	0.767(0.620,0.913)	0.933	0.600	0.800
intermediate radiologist B+radiomics	0.817(0.683,0.950)	0.933	0.700	0.800
junior radiologist C	0.733(0.583,0 884)	0.867	0.600	0.740
junior radiologist C+radiomics	0.750(0.605,0.895)	0.800	0.700	0.740
Intermediate respiratory physician D	0.700(0.543,0.857)	0.900	0.500	0.740
Intermediate respiratory physician D+radiomics	0.775(0.632,0.918)	0.900	0.650	0.800
junior respiratory physician E	0.633(0.474,0.793)	0.667	0.600	0.640
junior respiratory physician E+radiomics	0.775(0.636,0.914)	0.800	0.750	0.780

Senior radiologist *A* (Zhiyong Li, with 29 years of diagnostic chest imaging experience), intermediate radiologist *B* (Ruyi Bao, with 15 years of diagnostic chest experience), junior radiologist *C* (Siyu Che, with 5 years of diagnostic chest experience), intermediate respiratory physician *D* (Yadong Zhao, with 16 years of diagnostic chest experience), junior respiratory physician *E* (Meihong Liu, with 4 years of diagnostic chest experience); *AUC*, area under the receiver operating characteristic curve; *CI*, confidence interval; *SEN*,sensitivity; *SPE*,specificity; *ACC*,accuracy.

After comprehensively considering the stability of the models in the training and test datasets and the balance of various indicators, we believed that the CM established by the LR method was the most stable (**Table 4**).

**Table 4:** Diagnostic efficacy of the CM based on five machine learning methods in the training and test datasets.

Model	Training dataset				Test dataset			
	AUC (95% CI)	SEN	SPE	ACC	AUC (95% CI)	SEN	SPE	ACC
LR	0.917(0 870, 0 965)	0.870	0.739	0.817	0.825 (0 698, 0 952)	0.833	0.800	0.820
SVM	0.891(0 837, 0 934)	0.913	0.652	0.809	0.830 (0 719, 0 925)	0.833	0.800	0.820
RF	0.976 (0 957, 0 992)	0.957	0.848	0.913	0.778 (0 656, 0 882)	0.733	0.750	0.740
DT	0.887 (0 837, 0 932)	0.913	0.630	0.800	0 729(0 600, 0 839)	0.833	0.500	0.700
KNN	1.000 (1 000, 1.000)	1.000	1.000	1.000	0 642(0 528, 0 754)	0.733	0.550	0.660

LR, logistic regression; SVM, support vector machine; RF, random forest; DT, decision tree; KNN, K-nearest neighbor; AUC, area under the receiver operating characteristic curve; CI, confidence interval; *SEN*,sensitivity; *SPE*,specificity; *ACC*,accuracy.

**Table 5** shows the summary of existing studies on enhanced CT radiomics approaches for discriminating between malignant and benign lesions.

**Table 5:** Summary of existing studies on enhanced CT radiomics approaches for discriminating between malignant and benign lesions.

References	Number of Cases	Lesion Type	Imaging modality	Diagnostic Performance of the best Radiomics Model
(Liu et al. 2020) [26]	210	pulmonary solid nodules (≤10mm)	enhanced CT	training set: AUC = 0.911 validation set: AUC = 0.857
(Xu et al. 2023) [29]	242	pulmonary solid nodules	enhanced CT	training set: AUC = 0.97 validation set: AUC = 0.97
(Zhao et al. 2023) [27]	128	solid nodules or masses	enhanced CT	training set: AUC = 0.948 validation set: AUC = 0.917
(Zhao et al. 2022) [30]	101	solid nodules or masses	enhanced CT	training set: AUC = 0.922 validation set: AUC = 0.833
(He et al. 2016) [12]	240	pulmonary solid nodules	enhanced CT	training set: AUC = 0.862 validation set: AUC = 0.750

AUC, area under the receiver operating characteristic curve

## Discussion

In this study, the radiomics models we developed outperformed the CIM in the differential diagnosis of benign and lung adenocarcinoma lesions presenting as larger solid nodules and masses. The ERM showed a greater advantage over the PRM. Moreover, the CM emerged as the best model. The AUCs of both the radiologists and respiratory physicians improved after the radiomics model was utilized, further elucidating the value of the radiomics model.

First, the innovation of this study is that the size of the lesions are large solid nodules and masses. Currently, we found 5 studies have developed enhanced radiomics models to distinguish between benign and malignant lesions. Among them, the inclusion criterion in one study [26] was nodules ≤10 mm in size. The inclusion criteria in two studies [12, 29] did not specify the lung nodule lesion sizes. In the other two studies [27, 30], the inclusion criteria in terms of the lesion size were nodules and masses. In our study, the inclusion criteria in terms of the lesion size were larger nodules and masses. According to the Lung-RADS guidelines, larger solid nodules have a higher probability of malignancy. Therefore, constructing a radiomics model using enhanced scanning to identify benign and lung adenocarcinoma lesions presenting as larger solid nodules and masses may be more clinically meaningful. In addition, the novelty of this study lies in the exclusion of hamartomas from the included lesions. Liu et al. and He et al. have shown that hamartomas are among the included lesions [12, 26]. The inclusion criteria in Xu et al. studies did not indicate whether or not to include hamartoma [29]. In our study, hamartomas were explicitly excluded from the included lesions because hamartomas are lesions with obvious benign features. Moreover, tuberculosis with large calcifications can be diagnosed as benign lesions on plain CT alone, and our study did not include such lesions. Our study included cases that were difficult to identify by plain CT scans alone and required the use of contrast-enhanced scans for further differential diagnosis. The results obtained in such cases may be more clinically helpful.

The diagnostic efficacy of the ERM was superior to that of the PRM. A recent study on the differentiation of lung tuberculosis and adenocarcinoma demonstrated that enhanced CT images (training set AUC = 0.933, test set AUC = 0.881) have better diagnostic performance than plain CT images (training set AUC = 0.861, test set AUC = 0.756) [27], which was consistent with the results of our study. Enhanced scanning can identify lesions that are not detectable via plain scanning, and the status of the blood supply to the lesion can be determined [24, 25]. After the concentration of iodine increases in the blood, the concentration of iodine in the organ and the lesion can differ, resulting in a density difference, which is also conducive to the detection and diagnosis of the lesion. Therefore, enhanced scans are more helpful than plain scans in identifying benign and malignant lesions. These factors may also explain why the ERM displayed an increased AUC.

In this study, the CM provided added value in the differential diagnosis of benign and lung adenocarcinoma larger solid nodules and masses. Perandini et al. [31] compared the performance of four predictive models for the risk assessment and decision analysis of 285 cases of isolated pulmonary nodules. The four models were the Mayo, Gurney, PKUPH and BIMC models, and the AUC values of the four models were 0.775, 0.794, 0.889, and 0.898, respectively. The predictive ability of the BIMC model was better for isolated pulmonary nodules. In this study, the predictive ability of our CM was better, with AUCs of 0.917 and 0.825 for the training and test datasets, respectively. The predictive ability of the CM model was better for larger solid nodules and masses.

The diagnostic efficacy varied among doctors with different seniority levels and specialties (AUCs ranging from 0.633–0.867). Empirical medicine plays an important role in clinical work, and different specialties can also affect clinical diagnosis. The diagnostic stability among different doctors was lower than that of the radiomic model. In the test dataset, the AUCs of the ERM and CM all exceeded 0.800.

The AUC of the CM (0.825) was equivalent to that of the senior radiologist (0.867), and was better than those of the intermediate and junior radiologists (AUC = 0.767 and AUC = 0.733, respectively) and the intermediate and junior respiratory physicians (AUC = 0.700 and AUC = 0.633, respectively). Hu et al. [32] used an integrated model to differentiate pulmonary cryptococcosis nodules from lung adenocarcinomas. The integrated model achieved the highest AUC of 0.801, which was greater than that of the junior radiologist (AUC = 0.689) but was not significantly different from that of the senior radiologist (AUC = 0.784). These findings are consistent with the results of our study.

Three radiologists and two respiratory physicians improved their initial AUC after referring to the radiomics model results. A study by Horvat et al. [33] developed and externally validated a combined model, which increased the diagnostic performance of the junior radiologist (the specificity increased from 90% to 93%) in predicting pathological complete response after neoadjuvant treatment in patients with locally advanced rectal cancer. These findings are consistent with the results of our study (after using the radiomics model, the specificity of the junior radiologist specificity increased from 60% to 70%, and that of the junior respiratory physician increased from 60% to 75%). Therefore, we infer that if doctors apply the CM in their clinical work, it may help in the diagnosis and treatment of patients.

The current study has several limitations. First, this study was retrospective, so the data collected from patients could be potentially biased. Second, owing to the lack of arterial and delayed phase scans, only one phase of the enhanced scans was selected to assess the enhanced CT images in this study, and more phases need to be assessed in the future. Third, different CT scanners were used in this study. The use of several different CT scanners may have affected the assessment of some of the CT findings due to partial volume effects. Fourth, an external validation group was lacking. Fifth, this study focused on benign and lung adenocarcinoma larger solid nodules and masses alone, and this lesion distribution bias might have affected the results. Sixth, several studies on the characterization of isolated pulmonary nodules or differentiation between benign and malignant lung nodules via deep learning have been reported, but deep learning was not employed in this paper.

## Conclusion

In conclusion, in this study, the CIM, PRM, ERM and CM were established for the differential diagnosis of benign and lung adenocarcinoma larger solid nodules and masses. The CM performed the best among these models. The diagnostic efficacy of three radiologists and two respiratory physicians improved after the application of radiomics model. This model can facilitate therapeutic decision-making and the management of benign and lung adenocarcinoma lesions presenting as larger solid nodules and masses in the clinical setting. However, more studies are needed to validate the performance of the combined model before it can be applied in a real-world setting.

## Supporting information

S1 Fig Model formulas for the *PRM* and the *ERM*. *PRM*, plain CT radiomics model; *ERM*, enhanced CT radiomics model.
(TIF)

S1 Table Clinical and imaging data. (XLSX)

S2 Table Radiomics plain data. (XLSX)

S3 Table Radiomics enhanced data. (XLSX)

S4 Table Univariable and multivariable analyses. *OR* odds ratio, *CI* confidence interval*P value < 0.05.
(XLSX)
