# Supplementary figures and images for: Differential diagnosis of benign and lung adenocarcinoma presenting as larger solid nodules and masses based on multiscale CT radiomics
Source: PLoS One. 2024 Oct 4;19(10):e0309033. doi: 10.1371/journal.pone.0309033 (PMC11451992; doi:10.1371/journal.pone.0309033)

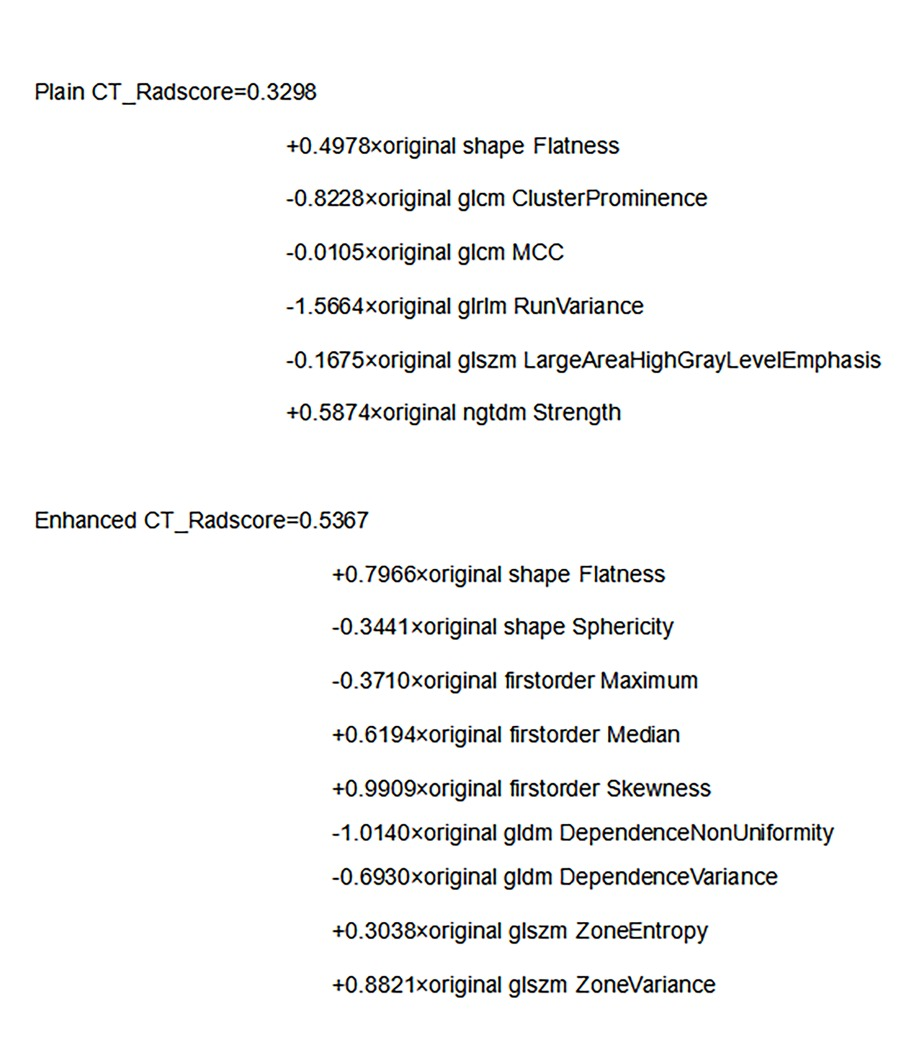

Supplement: S1 Fig — PRM, plain CT radiomics model; ERM, enhanced CT radiomics model. (TIF) [file pone.0309033.s001.tif]
